# Inhibitors of signal peptide peptidase and subtilisin/kexin-isozyme 1 inhibit Ebola virus glycoprotein-driven cell entry by interfering with activity and cellular localization of endosomal cathepsins

**DOI:** 10.1371/journal.pone.0214968

**Published:** 2019-04-11

**Authors:** Teresa Plegge, Martin Spiegel, Nadine Krüger, Inga Nehlmeier, Michael Winkler, Mariana González Hernández, Stefan Pöhlmann

**Affiliations:** 1 Infection Biology Unit, German Primate Center–Leibniz Institute for Primate Research, Göttingen, Germany; 2 Faculty of Biology and Psychology, University Göttingen, Göttingen, Germany; 3 Institute of Microbiology and Virology, Brandenburg Medical School Theodor Fontane, Senftenberg, Germany; 4 Institute of Virology, University of Veterinary Medicine Hannover, Hannover, Germany; 5 Research Center for Emerging Infections and Zoonoses, University of Veterinary Medicine Hannover, Hannover, Germany; Division of Clinical Research, UNITED STATES

## Abstract

Emerging viruses such as severe fever and thrombocytopenia syndrome virus (SFTSV) and Ebola virus (EBOV) are responsible for significant morbidity and mortality. Host cell proteases that process the glycoproteins of these viruses are potential targets for antiviral intervention. The aspartyl protease signal peptide peptidase (SPP) has recently been shown to be required for processing of the glycoprotein precursor, Gn/Gc, of Bunyamwera virus and for viral infectivity. Here, we investigated whether SPP is also required for infectivity of particles bearing SFTSV-Gn/Gc. Entry driven by the EBOV glycoprotein (GP) and the Lassa virus glycoprotein (LASV-GPC) depends on the cysteine proteases cathepsin B and L (CatB/CatL) and the serine protease subtilisin/kexin-isozyme 1 (SKI-1), respectively, and was examined in parallel for control purposes. We found that inhibition of SPP and SKI-1 did not interfere with SFTSV Gn + Gc-driven entry but, unexpectedly, blocked entry mediated by EBOV-GP. The inhibition occurred at the stage of proteolytic activation and the SPP inhibitor was found to block CatL/CatB activity. In contrast, the SKI-1 inhibitor did not interfere with CatB/CatL activity but disrupted CatB localization in endo/lysosomes, the site of EBOV-GP processing. These results underline the potential of protease inhibitors for antiviral therapy but also show that previously characterized compounds might exert broader specificity than initially appreciated and might block viral entry via diverse mechanisms.

## Introduction

Anthropogenic drivers including climate change, exploitation of natural resources and global travel promote the constant emergence of novel pathogens in the human population [1–3]. Emerging viral infections can be associated with significant morbidity and mortality. For instance, the outbreak and subsequent spread of a new bunyavirus, severe fever with thrombocytopenia syndrome virus (SFTSV) in China in 2009, was associated with more than 7,000 cases until 2016 and a case-fatality rate of up to 30% [4, 5]. Moreover, SFTS cases have recently also been reported in other Asian countries, including South Korea [6] and Japan [7]. Similarly, the first outbreak of Ebola virus disease in West Africa in 2013 unfolded into a epidemic with more than 10,000 deaths and almost 30,000 cases [8]. At present, there are no approved antiviral drugs against SFTSV, EBOV and several other emerging viruses. The targeting of host cell factors required for spread of these viruses but dispensable for cellular survival is considered a promising approach to antiviral therapy, since the respective drugs might exert broad antiviral activity (in case several viruses depend on the same factor) and might be associated with a high barrier against resistance development.

The glycoproteins of enveloped viruses facilitate viral entry into host cells. For this, they bind to host cell receptors and fuse the viral membrane with a cellular membrane, which allows delivery of the viral genetic information into the host cell cytoplasm [9]. However, viral glycoproteins are synthesized as inactive precursors and depend on priming by host cell proteases to transit into an active form [10, 11]. Priming can occur at different cellular localizations, for instance the constitutive secretory pathway, the plasma membrane and the extracellular space, and can proceed during different stages of viral spread, including glycoprotein biogenesis in infected cells, particle release from infected cells and entry into new target cells [10, 11]. Similarly, different host cell proteases can be hijacked by viruses for priming. For instance, the serine protease SKI-1 and other proprotein convertases are used by Lassa virus (LASV) [12] and human immunodeficiency virus (HIV) [13]), type II transmembrane serine proteases are used by influenza A viruses and coronaviruses [14–17] and the endo/lysosomal cysteine proteases cathepsin B and cathepsin L (CatB/CatL) are used by Ebola virus (EBOV) [18], Middle East respiratory syndrome coronavirus (MERS-CoV) [19, 20] and severe acute respiratory syndrome coronavirus (SARS-CoV) [21]). Thus, the activity of host cell proteases is required for priming of viral glycoproteins and for viral infectivity and the responsible enzymes constitute potential targets for antiviral intervention.

The order *Bunyavirales* comprises more than 350 agents, several of which are considered emerging and highly pathogenic. The glycoprotein precursor (termed Gn/Gc) of bunyaviruses is processed by signal peptidase in the endoplasmic reticulum of infected cells [22]. In addition, Crimean Congo hemorrhagic fever virus Gn/Gc is processed by proprotein convertases [23–25]. More recently, Shi and colleagues reported that Gn/Gc of Bunyamwera virus is also processed by signal peptide peptidase (SPP) [26], an intramembrane aspartyl protease that cleaves membrane associated signal peptides following their liberation from nascent proteins by signal peptidase. Here, we investigated whether SPP activity is also required for SFTSV-Gn/Gc to transit into an active state.

We report that inhibition of SPP and SKI-1 activity did not interfere with SFTSV Gn + Gc-driven host cell entry but, counterintuitively, blocked entry driven by the EBOV glycoprotein (GP). Furthermore, we show that the SPP inhibitor blocks EBOV-GP-driven entry by interfering with CatB activity while the SKI-1 inhibitor blocks entry by altering the cellular localization of CatB.

## Results

### SKI-1 and SPP inhibitors inhibit transduction mediated by EBOV-GP

The Bunyamwera orthobunyavirus Gn/Gc has been shown to be processed by SPP [26]. Therefore, we investigated whether SPP is also involved in the processing of the Gn/Gc of the highly pathogenic SFTSV. For this, we used rhabdoviral pseudotypes bearing SFTSV-Gn/Gc [27] and the SPP inhibitor (Z-LL)_2_ ketone. As negative control, we employed a SKI-1 inhibitor, PF-429242, since SKI-1 was shown not to be involved in SFTSV Gn/Gc processing [27]. In contrast, SKI-1 processes the Lassa virus glycoprotein precursor (LASV-GPC) in infected cells and processing is essential for viral infectivity [12]. Therefore, LASV-GPC bearing pseudotypes were included as control for the inhibitory activity of PF-429242 in our study. Finally, we included rhabdoviral pseudotypes bearing vesicular stomatitis virus glycoprotein (VSV-G) and Ebola virus glycoprotein (EBOV-GP) in our analyses, since processing of these glycoproteins and entry driven by these glycoproteins should be independent of SKI-1 and SPP activity.

**Fig 1:**
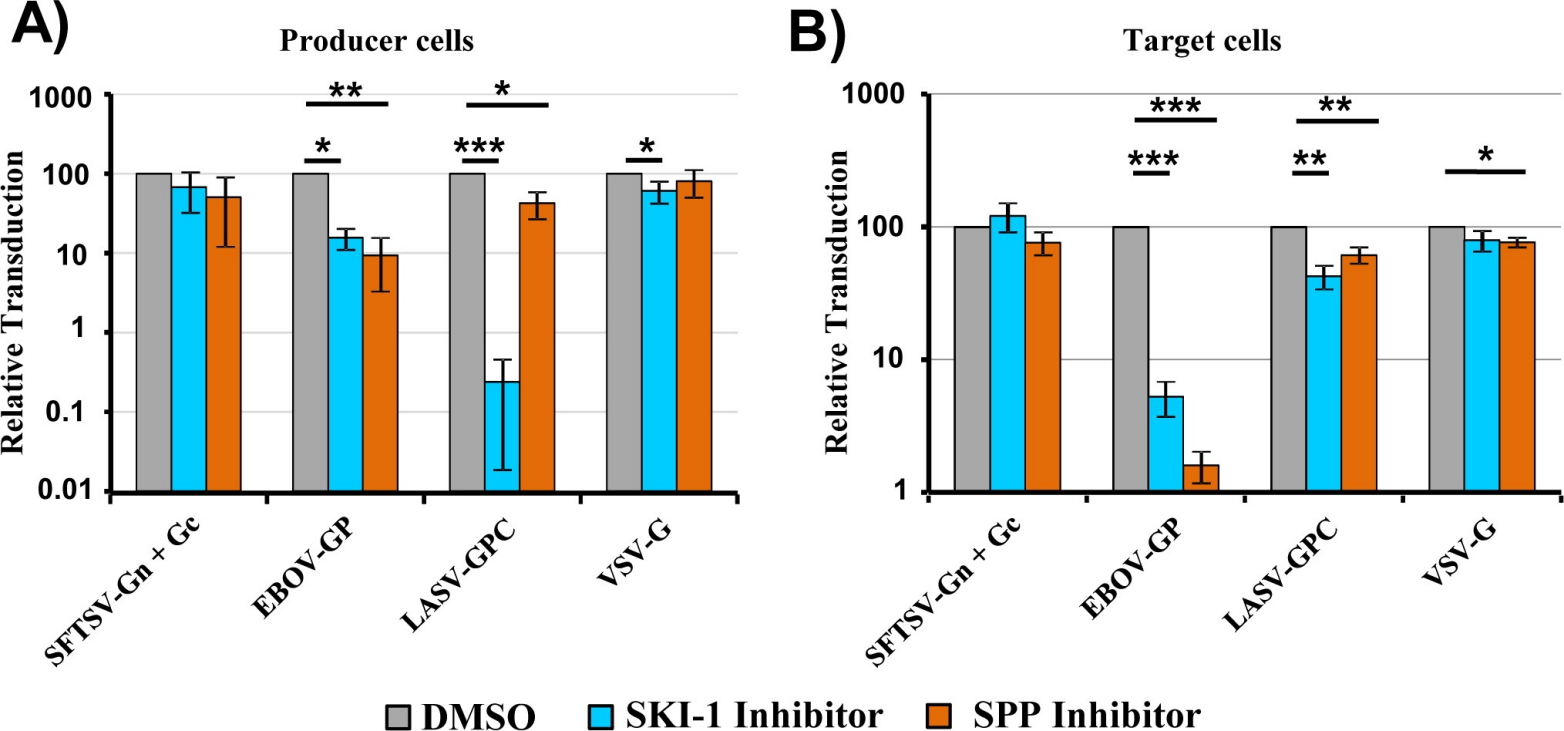
SKI-1 and SPP inhibitors block transduction mediated by EBOV-GP. (A) Rhabdoviral vectors encoding luciferase and pseudotyped with the indicated viral glycoproteins were produced in 293T cells in the presence of an SKI-1 inhibitor (PF429242, 30 μM), an SPP inhibitor ((Z-LL)_2_-ketone, 100 μM) or DMSO as a control. Protease inhibitor treatment started 6 h post transfection of the glycoprotein expression plasmids. Culture supernatants containing the rhabdoviral vectors were then used for transduction of target 293T cells. Transduction efficiency was determined by quantifying luciferase activities in cell lysates at 24 h post transduction. Transduction mediated by vectors produced in the presence of DMSO was set as 100%. (B) Rhabdoviral vectors pseudotyped with the indicated viral glycoproteins were used for transduction of target 293T cells in the presence of the indicated inhibitors or DMSO as a control. Protease inhibitor treatment was started 24 h prior to transduction with pseudotyped rhabdoviral vectors using the same inhibitor concentrations as depicted in (A). Transduction efficiency was determined by quantifying luciferase activities in cell lysates at 24 h post transduction. Transduction mediated by vectors produced in the presence of DMSO was set as 100%. The average of at least three independent experiments performed with triplicate samples is shown in panels A and B. Error bars indicate standard error of mean (SEM). Asterisks indicate a statistical significant decrease (* = p<0.05; ** = p<0.01; *** = p<0.001) of transduction efficiency relative to the DMSO control.

Host cell proteases can process viral glycoproteins in infected cells and during viral entry into target cells. In order to mimic glycoprotein processing in infected cells, we produced SFTSV-Gn/Gc, VSV-G, LASV-GPC and EBOV-GP bearing pseudotypes in the presence and absence of non-cytotoxic concentrations of SPP and SKI-1 inhibitor. We found that infectivity of VSV-G bearing pseudotypes was not markedly modulated by SPP and SKI-1 inhibitors, as expected, although a slight reduction of infectivity was observed in the presence of SKI-1 inhibitor (Fig 1A). Similarly, SKI-1 and SPP inhibitors did not appreciably modulate infectivity of SFTSV-Gn/Gc pseudotypes (Fig 1A). In contrast, the SKI-1 inhibitor markedly reduced infectivity of LASV-GPC-bearing pseudotypes while the SPP inhibitor had only a modest effect. Finally, and to our surprise, infectivity of EBOV-GP-bearing pseudotypes was markedly reduced by SPP and SKI-1 inhibitors (Fig 1A).

In order to mimic glycoprotein processing during viral entry, target cells were preincubated with SKI-1 and SPP inhibitor before addition of pseudotypes. Again, no marked effect was observed for VSV-G- and SFTSV-Gn/Gc-bearing pseudotypes and only modest inhibition of LASV-GPC-driven entry was observed (Fig 1B). In contrast, preincubation of target cells with SPP and SKI-1 inhibitor strongly reduced EBOV-GP-driven entry (Fig 1B). Notably, a more profound inhibition of EBOV-GP-mediated entry was observed when target cells were preincubated with SPP and SKI-1 inhibitors as compared to incubation of particle producing cells (Fig 1). Thus, SPP and SKI-1 inhibitor might mainly exert their antiviral activity during EBOV-GP-driven entry. Collectively, these results indicate that activity of SPP and SKI-1 in infected cells and in target cells may not be required for SFTSV and VSV infectious entry. Moreover, our results agree with the finding that LASV-GPC processing by SKI-1 in infected cells is required for viral infectivity. Finally, our results suggest that activity of SKI-1 and SPP or related proteases sensitive to the SKI-1 and SPP inhibitors might be required for EBOV-GP-driven host cell entry.

### SKI-1 and SPP inhibitors block cell entry driven by diverse filovirus glycoproteins and blockade occurs at one hour post exposure of virus to cells

**Fig 2:**
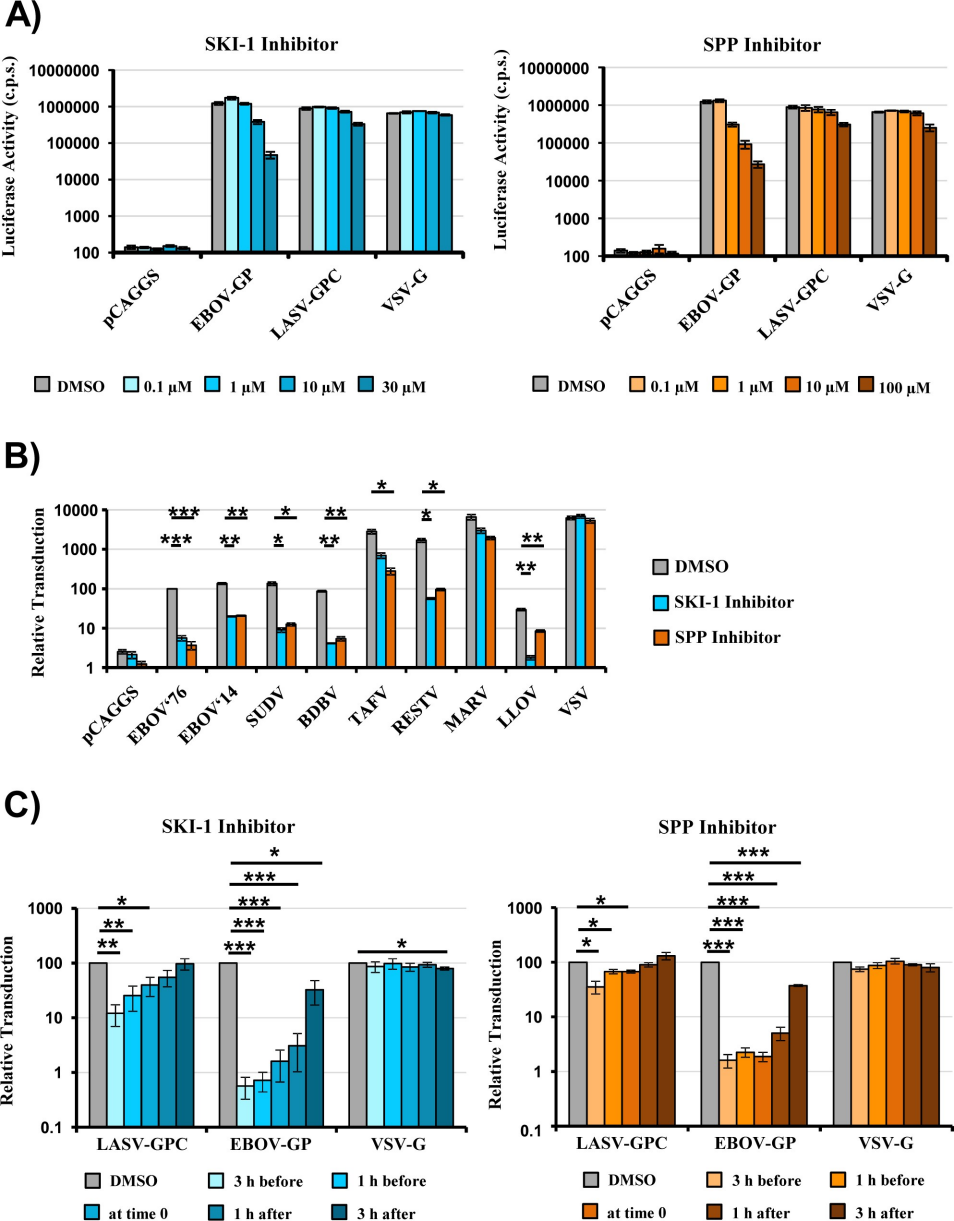
SPP and SKI-1 inhibitors block entry driven by diverse filovirus glycoproteins in a concentration and time-dependent manner. (A) 293T cells were incubated with the indicated inhibitors or DMSO as control and transduced with rhabdoviral particles bearing the indicated viral glycoproteins. Transduction efficiency was analyzed by determining luciferase activities in cell lysates at 24 h post transduction. The results of a representative experiment performed with triplicate samples are shown. Error bars indicate standard deviation. Similar results were obtained in a separate experiment (SD). C.p.s., counts per second. (B) Rhabdoviral vectors pseudotyped with the indicated filovirus glycoproteins were used for transduction of target 293T cells pretreated with the indicated inhibitors or DMSO as a control. Transduction efficiency was analyzed by determining luciferase activities in cell lysates at 24 h post transduction. The average of three independent experiments performed with triplicate samples is shown. Transduction mediated by particles bearing EBOV-GP (GP from Mayinga variant, EBOV’76) was set as 100%. Error bars indicate standard error of the mean (SEM). (C) 293T cells were treated with the indicated inhibitors at the indicated time points and transduced with rhabdoviral vectors pseudotyped with the indicated viral glycoproteins. Transduction efficiency was analyzed by determining luciferase activities in cell lysates at 24 h post transduction. The average of three independent experiments performed with triplicate samples is shown. Transduction measured in the presence of DMSO was set as 100%. Error bars indicate standard error of mean (SEM). Asterisks indicate a statistical significant decrease (* = p<0.05; ** = p<0.01; *** = p<0.001) of transduction in the presence of inhibitor as compared to DMSO.

We next explored whether the inhibitory effect of SPP and SKI-1 inhibitors on EBOV-GP-driven entry was concentration dependent and was also observed for glycoproteins from other viruses of the *Filoviridae* family. Moreover, we investigated whether an early or a late step of the entry process was blocked. Preincubation of target cells with SKI-1 and SPP inhibitor reduced EBOV-GP-driven entry in a concentration-dependent fashion but had little effect on entry driven by LASV-GPC and VSV-G (Fig 2A). Moreover, exposure of target cells to SKI-1 and SPP inhibitors reduced transduction mediated by the glycoprotein of the EBOV responsible for the West African Ebola virus disease epidemic (EBOV 2014) and the glycoproteins of other ebolavirus species (Fig 2B). Inhibition of entry driven by the glycoprotein of Lloviu virus (LLOV), the only member of the *Cuevavirus* genus within the *Filoviridae* family, was also observed (Fig 2B). In contrast, entry driven by the glycoprotein of Marburg virus (MARV) was only modestly inhibited. Finally, a time-of-addition experiment revealed that SKI-1 and SPP inhibitors efficiently blocked EBOV-GP- but not LASV-GPC- or VSV-G-driven entry when added to target cells before and during exposure to pseudotypes but started to lose inhibitory activity when added at 1 h after the addition of pseudotypes (Fig 2C). Collectively, these results show that SKI-1 and SPP inhibitors block host cell entry driven by most but not all filovirus glycoproteins. Moreover, they suggest that a process of the entry cascade is blocked that commences roughly at 1 h after virus binding to cells.

### SPP and SKI-1 inhibitors also block SARS-S- and MERS-S-driven entry

**Fig 3:**
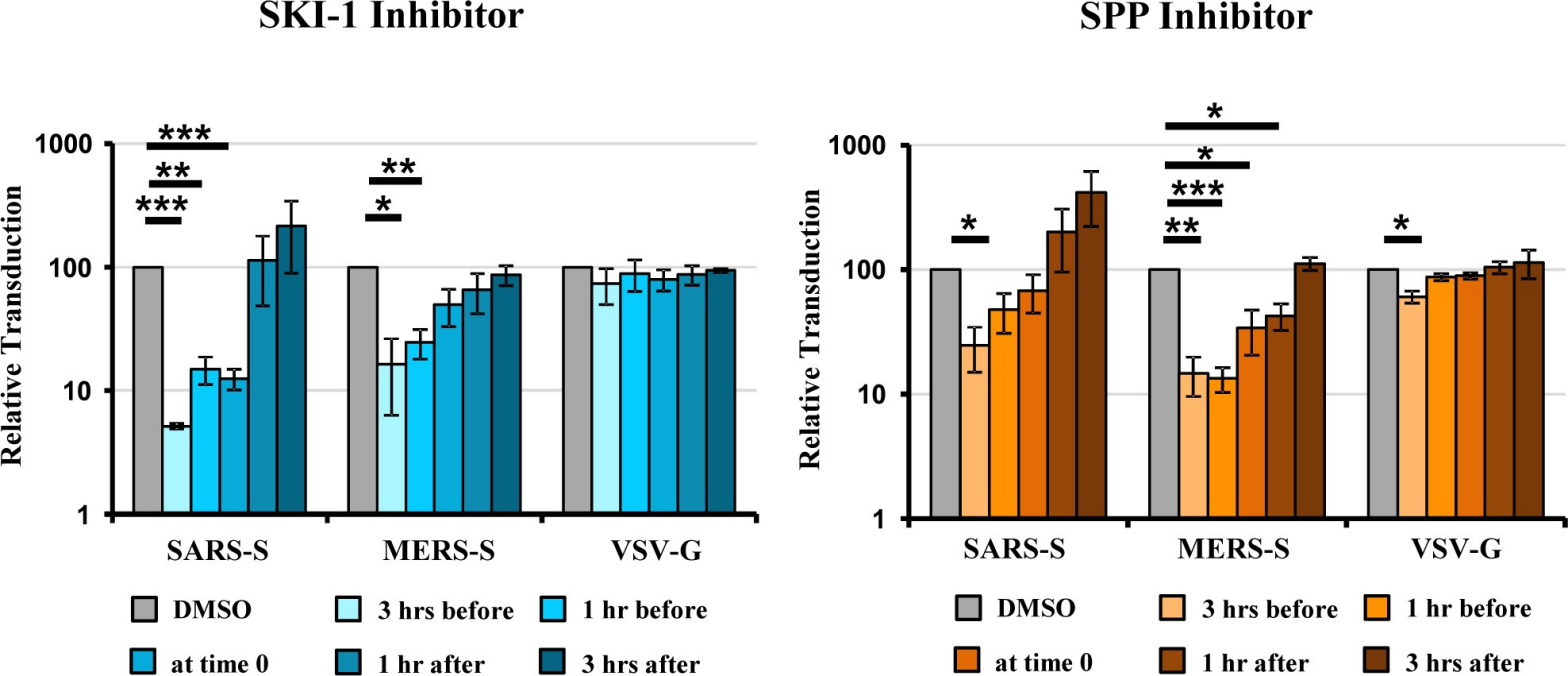
SPP and SKI-1 inhibitors block SARS-S- and MERS-S-driven entry. Rhabdoviral particles pseudotyped with the indicated viral glycoproteins were used for transduction of 293T cells treated with the indicated inhibitors at the indicated time points. Transduction efficiency was analyzed by determining luciferase activities in cell lysates at 24 h post transduction. The average of three independent experiments performed with triplicate samples is shown. Transduction measured in the presence of DMSO was set as 100%. Error bars indicate standard error of mean (SEM). Asterisks indicate a statistical significant decrease (* = p<0.05; ** = p<0.01; *** = p<0.001) of transduction efficiency in the presence of inhibitor compared to DMSO.

The cellular proteases CatB and CatL process EBOV-GP in late endosomes/lysosomes and thereby allow GP binding to the intracellular receptor NPC1, which is essential for infectious viral entry [28–30]. It can be inferred from previous work that processing of GP by CatB/CatL might occur roughly at one hour after virus binding to target cells [30], suggesting that SKI-1 and SPP inhibitors might block this process and thereby inhibit EBOV-GP-mediated entry. In order to explore this possibility, we investigated whether SKI-1 and SPP inhibitor blocked cellular entry driven by the spike proteins of MERS-CoV and SARS-CoV (MERS-S, SARS-S), which also depends on CatL activity in some cell types [19–21]. SKI-1 and SPP inhibitor had only a modest if any effect on VSV-G-driven entry but inhibited MERS-S- and SARS-S-driven entry efficiently, and a time-of-addition analysis revealed that an early step in the entry process was blocked (Fig 3), most likely proteolytic priming of the S proteins.

### SPP inhibitor blocks CatL and CatB activity

**Fig 4:**
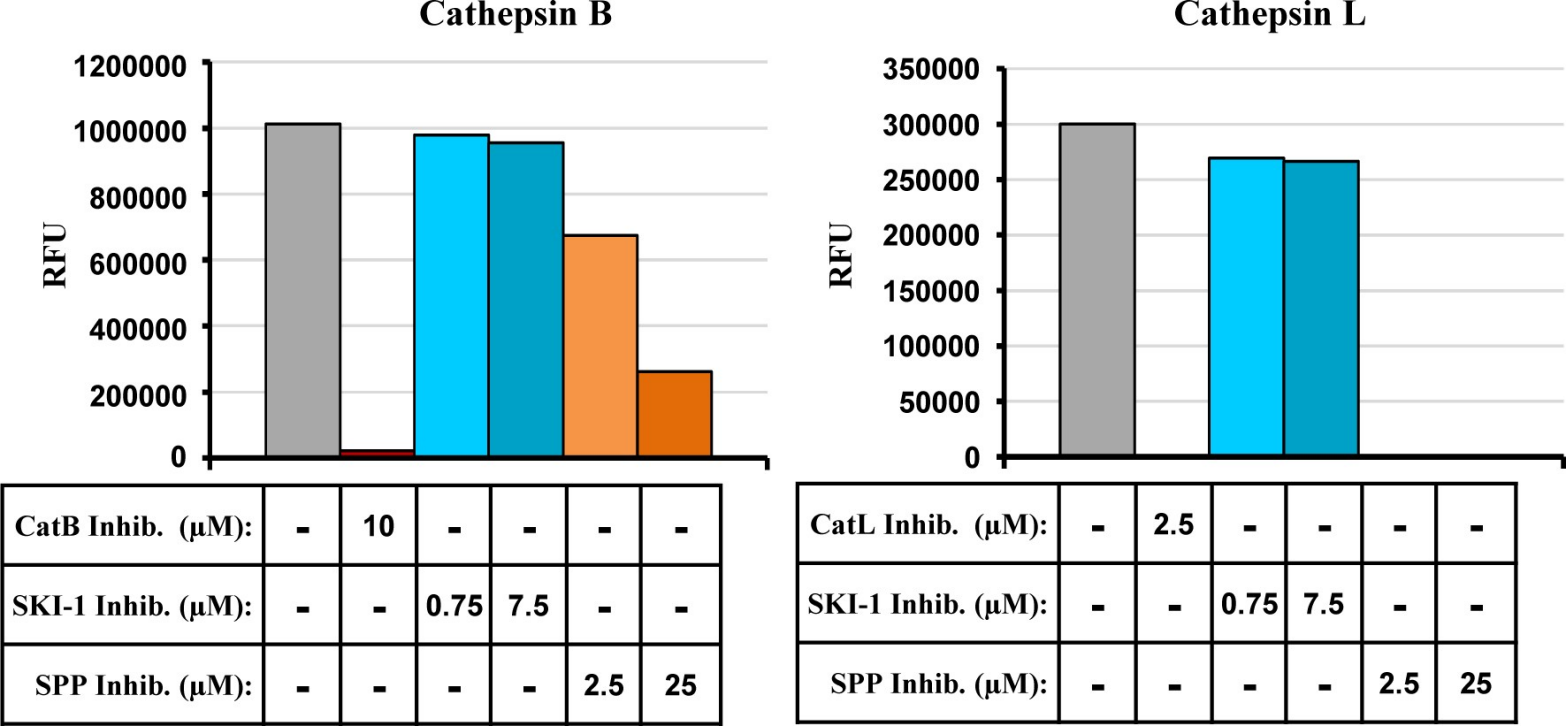
SPP inhibitor blocks the enzymatic activity of recombinant CatB and CatL. Cathepsin activities were determined by measuring the release of the fluorophore 7-amino-4-methylcoumarin (AMC) from peptide substrates Z-Arg-Arg-AMC (CatB) and Z-Phe-Arg-7-AMC (CatL). The results of a single representative experiment performed with duplicate samples are shown and were confirmed in a separate experiment. CatB inhibitor: CA074; CatL inhibitor: Z-FY(t-Bu)-DMK; RFU, relative fluorescence units.

Our results so far suggested that SPP and SKI-1 inhibitor might block EBOV-GP-mediated entry by inhibiting GP processing by CatB and CatL. We next investigated whether SPP and SKI-1 inhibitor indeed interfere with the proteolytic activity of CatL and CatB. For this, we employed commercial assay systems, which measure processing of a model substrate by recombinant CatB and CatL, respectively. The SKI-1 inhibitor had no appreciable effect on CatB or CatL activity (Fig 4), which was abrogated by inhibitors included as positive controls in the assay systems. In contrast, SPP inhibitor abolished CatL activity entirely and decreased CatB activity in a concentration dependent fashion (Fig 4). These results indicate that the SPP inhibitor blocks EBOV-GP-mediated entry by directly interfering with CatB/CatL activity.

### SKI-1 inhibitor interferes with processing of the proforms of CatB and CatL and alters the cellular localization of CatB

**Fig 5:**
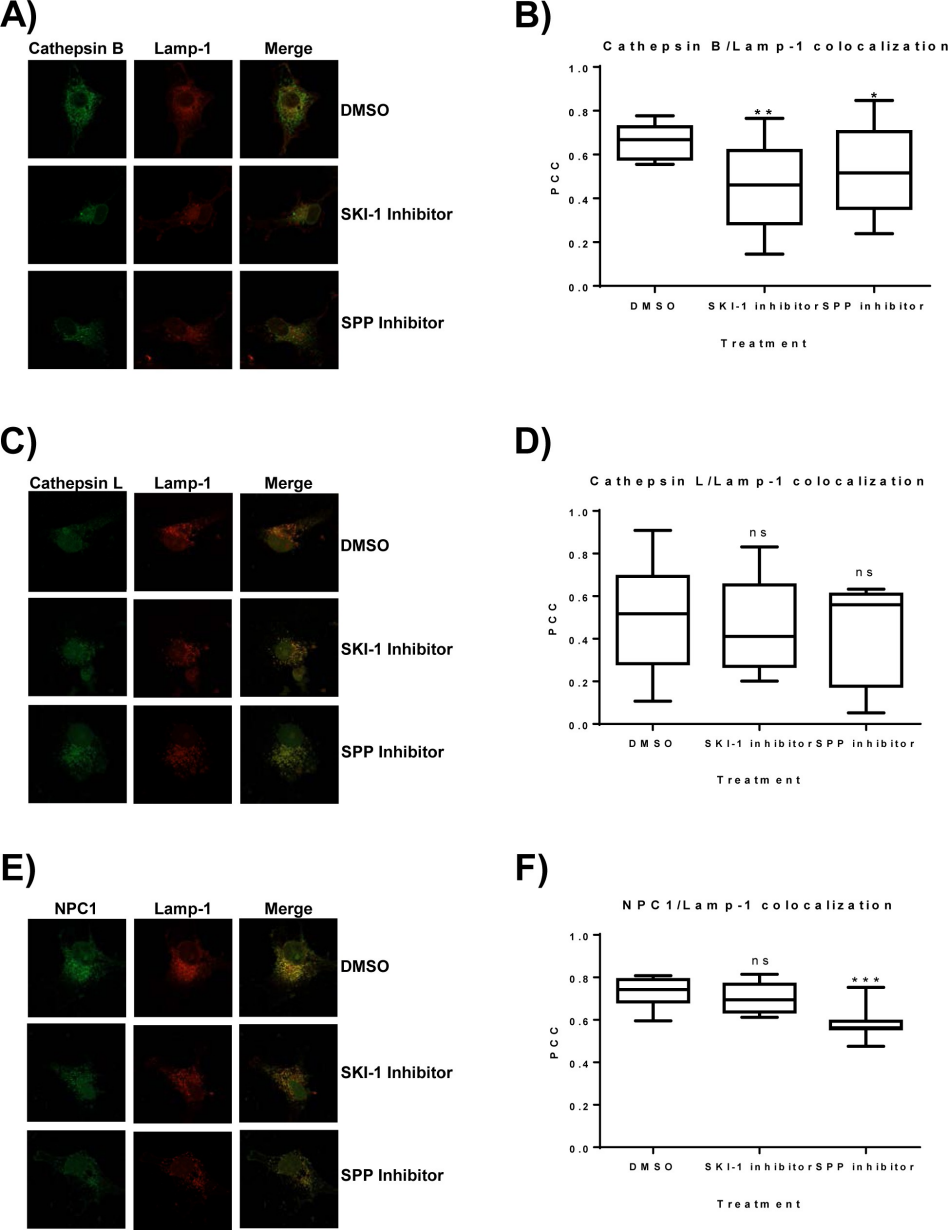
SKI-1 inhibitor disrupts the colocalization of CatB with Lamp-1. COS-7 cells were seeded on glass coverslips and cotransfected with plasmids encoding CatB, CatL or NPC1 all carrying a FLAG-tag and the late endosome/lysosome marker protein Lamp-1 fused to mScarlet. At 4 h post transfection, cells were treated with the indicated inhibitor and incubated at 37°C for 24 h after which cells were fixed and protein localization was analyzed by confocal immunofluorescence. Fluorescence images were taken at 63x magnification. Similar results were obtained in four independent experiments for CatB and CatL and in two independent experiments for NPC1. (A, C, E) Representative images for the colocalization of CatB, CatL and NPC1 with Lamp-1 in the presence of the indicated inhibitors. (B, D, F) Quantification of colocalization. Ten cells for each condition were selected and the Pearson Correlation Coefficient (PCC) for the overlap of CatB, CatL and NPC1-specific signals with Lamp-1 specific signals in single cells was determined. Asterisks indicate a statistically significant decrease (* = p<0.05; ** = p<0.01; *** = p<0.001) in colocalization of the indicated proteins with the lysosomal marker Lamp-1 in the presence of inhibitor in comparison to DMSO.

We had found that the SKI-1 inhibitor blocked EBOV-GP-driven entry most likely at the stage of proteolytic priming of GP by CatB/CatL without interfering with the activity of these proteases. In order to elucidate the mechanism underlying inhibition, we first asked whether SKI-1 inhibitor interferes with the conversion of CatB/CatL proenzymes into their catalytically active single and double chain forms. For this, we used cells transfected to express CatB/CatL. Immunoblot analysis of these cells revealed three prominent bands in the range between 30 and 45 kDa and a faint band of approximately 25 kDa, which likely represent the proform, a processing intermediate or glycoform, the single chain form and the heavy chain part of the double chain form (S1 Fig). Incubation of the CatB/CatL transfected cells with SKI-1 inhibitor at a final concentration of 30 μM for 24 h inhibited processing of the CatB and CatL proforms into the enzymatically active forms. Thus, production of the CatB/CatL single chain forms was inhibited and, in case of CatL, also the generation of the heavy chain part of the double chain form was blocked while the signals observed for the CatB heavy chain part of the double chain form were too faint to allow interpretation (S1 Fig). In contrast, the SPP inhibitor did not reduce production of the enzymatically active forms of CatB/CatL under the same conditions, but generally reduced CatB expression when tested at the highest concentration (S1 Fig). The conversion of CatB/CatL proenzymes into their active forms occurs in the lysosome. Therefore, we asked whether the SKI-1 inhibitor altered the cellular localization of CatB/CatL. For this, Cos7 cells were cotransfected with plasmids encoding Lamp-1, a cellular protein known to reside in the late endosome/lysosomal compartment, and either CatB, CatL or NPC1 and subsequently treated with the SKI-1 inhibitor, SPP inhibitor or DMSO as a control. Extensive colocalization of CatB, NPC1 and, to a lesser degree, CatL with Lamp-1 was observed (Fig 5), in agreement with published data. Notably, SKI-1 inhibitor treatment markedly disrupted the colocalization of CatB and Lamp-1 (Fig 5A and Fig 5B) while the SPP inhibitor had only a moderate but still statistically significant effect (Fig 5A). In contrast, none of the inhibitors interfered with colocalization of CatL with Lamp-1 (Fig 5C and Fig 5D) and, unexpectedly, the SPP inhibitor reduced NPC1 colocalization with Lamp-1 (Fig 5E and Fig 5F). Although confirmatory experiments with endogenously expressed proteins are pending, these results suggest that SKI-1 inhibitor blocks EBOV-GP-driven entry by reducing CatB localization in late endosomes/lysosomes, the site of EBOV-GP-driven membrane fusion.

## Discussion

Inhibition of host cell proteases responsible for processing of viral glycoproteins is an attractive approach to antiviral therapy. Such inhibitors might be particularly useful for treatment of emerging, highly pathogenic viruses against which no other antiviral options are available. Our results show that inhibitors of the host cell proteases SPP and SKI-1 do not impact SFTSV Gn + Gc-driven entry but block entry mediated by EBOV-GP. Moreover, we demonstrate that the SPP inhibitor blocks the enzymatic activity of the EBOV-GP processing host cell proteases CatB/CatL while the SKI-1 inhibitor interferes with localization of CatB in late endosomes/lysosomes, the site of EBOV-GP processing. Our results show that inhibition of SKI-1 blocks EBOV-GP-driven entry but also indicate that inhibitors of viral glycoprotein processing host cell proteases can interfere with viral entry in a direct and an indirect manner.

We had previously provided evidence that production of mature SFTSV Gc protein requires processing of the Gn/Gc precursor at an internal signal peptide sequence located at the N-terminus of mature Gc [27]. Shi and colleagues have demonstrated that processing of Gn/Gc of another Bunyavirus, Bunyamwera orthobunyavirus, depends on the activity of SPP [26]. Thus, SPP processes NSm, which is part of the Bunyamwera orthobunyavirus Gn/Gc, and processing is required for fusion of infected cells with uninfected cells [26]. For bunyaviruses like SFTSV, which do not possess an internal NSm between Gn and Gc, one may speculate that SPP processes the C-terminal portion of mature Gn proteins that remains in the membrane after separation of Gn and Gc by signal peptidase-mediated cleavage. This triggered us to investigate whether SPP is also required for production of functional SFTSV Gn + Gc, i.e. Gn + Gc that is capable of mediating host cell entry. We included a SKI-1 inhibitor in our studies, since we previously reported that this compound does not interfere with SFTV-Gn/Gc maturation and function [27], although it is well known to block LASV-GPC processing and viral infectivity [12]. Finally, we examined EBOV-GP-driven entry in our study, since entry driven by this glycoprotein should be independent of SKI-1 and SPP activity but should depend on GP processing by CatB/CatL [18]. Unexpectedly, we found that both SPP and SKI-1 inhibitor blocked EBOV-GP- but not SFTSV Gn + Gc-driven entry, raising questions regarding the underlying mechanism.

A time-of-addition experiment revealed that both SPP and SKI-1 inhibitor blocked a process starting approximately one hour after addition of virus to cells, which coincides with the time point previously determined for transport of EBOV-GP-bearing particles into NPC1^+^ compartments that express high levels of the GP processing proteases CatB/CatL [30]. Subsequently, several lines of evidence were obtained supporting the concept that SPP and SKI-1 inhibitors block EBOV-GP-driven entry by inhibiting CatB/CatL. First, SARS-S- and MERS-S-driven entry, which is known to be dependent on CatL activity under the conditions examined here [19–21], was blocked by SPP and SKI-1 inhibitors. Second, directed expression of TMPRSS2, which processes SARS-S and MERS-S at the cell surface and renders SARS-S- and MERS-S-mediated entry CatL-independent [15–17, 20], also abrogated the entry blockade installed by SPP and SKI-1 inhibitors (not shown). Third, entry driven by the glycoproteins of Lloviu virus and all ebolaviruses tested, which is known to depend on CatB/CatL activity although to different extends [18, 31–33], was also blocked by SPP and SKI-1 inhibitors. Fourth, MARV-GP-mediated entry, which is relatively CatB/CatL-independent [32, 33], was not efficiently blocked by CatB/CatL inhibitors.

Direct evidence for SPP and SKI-1 inhibitors interfering with CatB/CatL activity was obtained when we examined cleavage of artificial substrates, using commercial assay systems. These results showed that SPP inhibitor blocked activity of recombinant CatB and CatL, which are both cysteine proteases. This finding is in contrast to commercial sources offering (Z-LL)_2_ ketone as a SPP specific inhibitor. However, (Z-LL)_2_ ketone was originally described as a novel cysteine protease inhibitor [34] and only subsequent experiments revealed that it inhibits SPP, which is a presenilin-type aspartyl protease [35]. Thus, it is conceivable that some results obtained within previous studies examining SPP activity using (Z-LL)_2_ ketone as specific SPP inhibitor might need to be reinterpreted.

The SKI-1 inhibitor did not interfere with the activity of recombinant CatB/CatL, suggesting that the compound might block EBOV-GP processing by these proteases in an indirect fashion. SKI-1 plays a central role in cholesterol metabolism, since it cleaves and thereby activates sterol regulatory element binding proteins (SREBPs), which in turn control expression of several enzymes intimately involved in cholesterol synthesis [36]. Thus, SKI-1 inhibitor might interfere with EBOV-GP-driven entry by modulating cholesterol homeostasis. However, addition of cholesterol did not rescue EBOV-GP-driven from blockade by SKI-1 inhibitor (not shown), in keeping with the recent finding that inhibition of cholesterol synthesis does not interfere with GP-driven entry but blocks GP glycan maturation [37].

Apart from its role in cholesterol metabolism, SKI-1 is responsible for the generation of enzymatically active GlcNAc-1-phosphotransferase (GNPTAB) from a precursor protein [38]. Mature GlcNAc-1-phosphotransferase is in turn required for the generation of N-glycans with mannose 6 phosphate (M6P) residues, which serve as a lysosomal targeting signal for CatB/CatL [39] and many other cellular proteins. This raised the question whether blockade of SKI-1 activity might result in mislocalization of CatB. Such a scenario could explain why SKI-1 inhibitor blocked CatB processing, which is known to occur in lysosomes and to be required for enzymatic activity, as well as EBOV-GP-driven entry by. Confocal microscopy indeed revealed that SKI-1 inhibitor treatment reduced colocalization of CatB with Lamp-1, a protein targeted to endosomal/lysosomal membranes independent of the M6P pathway [40, 41]. Thus, SKI-1 most likely inhibits EBOV-GP-mediated entry by reducing the amounts of CatB in late endosomes/lysosomes, the site of GP priming. This scenario is in agreement with a recent study by Flint and colleagues who reported that GNPTAB expression is required for production of enzymatically active CatB and for EBOV-GP-driven host cell entry [42].

We observed that SKI-1 inhibitor blocked processing of both CatB and CatL and this is again consistent with the study by Flint and coworkers [42]. In the light of these findings we would have expected the SKI-1 inhibitor to interfere with the lysosomal localization of both CatB and CatL but altered lysosomal localization of CatL was not detected. However, CatL colocalization with the lysosomal marker Lamp-1 in untreated cells was already lower than that measured for CatB (compare PCC values in Fig 5), in keeping with published data [43, 44], and our assay might not have been sensitive enough to detect disruption of colocalization by SKI-1 inhibitor. It is noteworthy that a minor but statistically significant reduction of CatB/Lamp-1 colocalization was also observed upon treatment of cells with the SPP inhibitor and this inhibitor also markedly interfered with NPC1/Lamp-1 colocalization. The reasons for these effects are at present unknown. However, it should be noted that SPP-L2A, a SPP homologue which is also sensitive to the SPP inhibitor (Z-LL)_2_ ketone [45], is localized in the membranes of late endosomes and lysosomes. Moreover, it has been shown that lack of SPP-L2A activity disturbs endosomal membrane trafficking and morphology [46], which might explain the reduced colocalization of CatB and NPC1 with Lamp-1 in the presence of the SPP inhibitor. Future studies should reveal whether blockade of SPP-L2A or CatB/CatL activity by SPP inhibitor accounts for the reduced colocalization of NPC1/Lamp-1, since this might impact interpretation of results obtained upon analysis of EBOV entry in the presence of CatB/CatL inhibitors.

Collectively, our results confirm that host cell proteases which prime viral glycoproteins are potential targets for antiviral intervention. However, they also underline that highly specific inhibitors are needed in order to avoid pleiotropic effects. Finally, our findings and a previous study [47] show that interference with the activity of cellular proteases might impact availability of other host factors required for virus infection and vice versa.

## Material and methods

### Cell culture

Human embryonic kidney 293 T cells, African green monkey COS-7 kidney cells and African green monkey Vero 76 kidney cells were maintained in Dulbecco’s modified Eagle’s medium (DMEM; PAN Biotech) supplemented with 10% fetal bovine serum (FBS; Biochrome), 100 U/ml penicillin and 100 μg/ml streptomycin (PAN Biotech), and 1% L-glutamine (PAN Biotech). The cells were grown in a humidified atmosphere at 37°C and 5% CO_2_.

### Plasmids

Expression plasmids encoding the glycoproteins of EBOV-GP’76 (EBOV from the 1976 outbreak in the Democratic Republic of Congo, Mayinga variant, GenBank accession number AF086833.2, [48]) and EBOV’14 (Ebola virus/H.sapiens-wt/SLE/2014/Makona-G3838 of the West African EVD epidemic, Makona variant, GenBank accession number KM233105.1, [48]), LASV-GPC [49], MERS-S [19], SARS-S (Frankfurt-1 isolate) [50], VSV-G [49], LLOV-GP [31], MARV-GP [51] and GPs of ebolaviruses have been described previously [52]. Similarly, expression plasmids encoding the SARS-CoV receptor ACE2 [53], the MERS-CoV receptor DPP4 [54] and the EBOV receptor NPC1 [28] have also been described. The coding sequence for CatB (Genbank accession numberXM_017013097.2 (Homo sapiens cathepsin B (CTSB), transcript variant X1, mRNA) carrying a silent mutation (nt: A420G)) and CatL (Genbank accession number XM_005251716.4 (Homo sapiens cathepsin L (CTSL), transcript variant X1, mRNA) were RT-PCR amplified from total RNA of A549 cells and were inserted into plasmid pCAGGS. For cloning of Lamp-1-Scarlet, pmScarlet-i-C1 (obtained from Addgene; published in [55]) was inserted into the vector pmCherry-N1via AgeI and Bsp1407I, resulting in plasmid pScarlet-i-N1. Next, the Lamp-1 sequence from pEYFP-Lamp-1 [56] was inserted into pScarlet-i-N1 via EcoRI and BamHI. The identity of all PCR amplified sequences was verified by automated sequence analysis.

### Protease inhibitors

For inhibition of cellular proteases, 30 μM of the SKI-1/S1P inhibitor PF-429242 dihydrochloride (Sigma-Aldrich, Taufenkirchen, Germany) and 100 μM SPP inhibitor (Z-LL)_2_ ketone (Merck KGaA, Darmstadt, Germany) were used. Inhibitors were diluted in DMSO as recommended by the manufacturers. Cytotoxicity assays (Promega, Madison, USA) were performed to confirm that the inhibitor concentrations used did not affect cell viability.

### Production of rhabdoviral pseudotypes

Production of rhabdoviral pseudotypes has been described previously [48]. Briefly, human embryonic kidney 293T cells were seeded in T25 cell culture flasks at 500,000 cells/flask and calcium-phosphate-transfected with 6 μg of the glycoprotein expression plasmid of interest. After a 6 h incubation period, the cells were washed twice with PBS and fresh culture medium and either a protease inhibitor (SKI-1 inhibitor PF429242 or SPP inhibitor (Z-LL)_2_ ketone) or DMSO was added. At 30 h post transfection, the cells were transduced at a MOI of 0.2 with a replication-defective vesicular stomatitis virus (VSVΔG) pseudotyped with VSV-G and encoding luciferase and green fluorescent protein (GFP). After 1 h, the cells were washed three times with PBS and fresh culture medium was added that contained a 1:1,000 dilution of I1 (an anti-VSV-G mouse hybridoma supernatant from CRL-2700; American Type Culture Collection) to neutralize any remaining VSV activity as well as fresh protease inhibitor or DMSO. Twenty-four hours post transduction, rhabdoviral pseudotypes were harvested from the culture supernatants via sterile-filtration through 0.45 μm filters and stored at -80°C until use.

### Inhibition of CatB and CatL processing by protease inhibitors

In order to investigate inhibition of CatB and CatL processing by SKI-1 and SPP inhibitor, 293T cells were transfected with CatB and CatL expression plasmids and incubated with inhibitors for 3 h or 24 h before harvest. Thereafter, cells were lysed in 2x sodium dodecyl sulfate (SDS) loading buffer and CatB/CatL expression analyzed by immunoblot using a previously described protocol [57]. For detection of CatB expression, a mouse monoclonal antibody raised against CatB purified from human liver (Abcam) was used at a dilution of 1:400. For detection of CatL expression, a rabbit polyclonal serum raised against CatL amino acids 200–300 (Abcam) was used at a dilution of 1:400. Detection of bound antibodies was achieved using horseradish peroxidase coupled goat anti-mouse or goat anti-rabbit secondary antibodies (Dianova) at a dilution of 1:5,000. Finally, expression of β-actin was detected using a rabbit polyclonal serum (Sigma Aldrich) at a dilution of 1:1,000.

### Inhibition of viral entry by protease inhibitors

Infectivity of rhabdoviral pseudotypes in the presence and absence of inhibitors was measured by transduction of 293T cells, which were seeded in 96-well plates at 30,000 cells/well. At 24 h post seeding, cells were incubated with 50 μl pseudotyped virus for 1 h and were subsequently treated with protease inhibitor as indicated in the figure legends. For analysis of SARS-S- and MERS-S-driven transduction, target cells were first transfected with 0.2 μg of plasmids encoding ACE2 or DPP4, respectively, in order to allow for efficient S protein-driven host cell entry. In all experimental settings, transduction efficiency was quantified at 24 h post transduction by determining luciferase activity in cell lysates using a commercially available beetle-juice luciferase kit (p.j.k., Kleinbittersdorf, Germany) and a Hidex Plate Chameleon luminometer.

### Measurement of CatB and CatL activity

In order to investigate the impact of the SKI-1 and SPP inhibitors on the activity of recombinant CatB and CatL, the release of the fluorophore 7-amino-4-methylcoumarin (AMC) from peptide substrates Z-Arg-Arg-AMC (CatB) and Z-Phe-Arg-7-AMC (CatL) was determined using commercially available kits (InnoZyme Cathepsin B and L Kit, both Merck KGaA, Darmstadt, Germany). As positive controls for the inhibition of recombinant CatB and CatL CA074 and Z-FY(t-Bu)-DMK were used. Fluorescence was quantified using a Hidex Sense reader.

### Immunofluorescence and confocal microscopy

COS-7 kidney cells were seeded on glass coverslips in 24-well cell culture plates at 40,000 cells/well and incubated for 24 h. Subsequently, the cells were cotransfected with Lipofectamine 2000 (Invitrogen, Carlsbad, USA) with 1.33 μg of plasmids encoding CatB, CatL or NPC1 carrying a FLAG-tag and 0.67 μg of plasmid pLamp1-Scarlet encoding the late endosomal/lysosomal marker protein Lamp-1 fused to mScarlet. At four hours post transfection, cells were washed twice with PBS and treated with fresh culture medium containing protease inhibitor or DMSO as control. At 24 h post inhibitor treatment, the glass coverslips were washed once with PBS followed by fixation with 4% paraformaldehyde for 10 min at room temperature (RT), followed by washing three times with PBS. Next, glass coverslips were incubated in 0.2% Triton X-100 in PBS for 10 min at RT and washed three times with PBS. In preparation for antibody staining, glass coverslips were blocked for 30 min at 37°C with PBS containing 0.5% Tween and 1% bovine serum albumin (BSA). The primary antibody mouse monoclonal IgG1 anti-FLAG M2 antibody (F1804) (Sigma-Aldrich, Taufenkirchen, Germany) was diluted 1:200 in PBS containing 0.5% Tween and 1% BSA and glass coverslips were placed upside down on 25 μl droplets of the antibody dilution and incubated at room temperature for 1 h. Glass coverslips were then washed three times with PBS and again incubated upside down on 25 μl droplets containing secondary antibody (Alexa fluor 488 donkey anti-mouse IgG (Life Technologies, Eugene, OR, USA)) diluted 1:400 in PBS supplemented with 0.5% Tween and 1% BSA at room temperature for 1 h in the dark. Glass coverslips were washed twice with PBS followed by two washes with distilled H_2_O prior to mounting onto a microscope slide with mowiol. Fluorescence microscopy was performed on a Zeiss LSM800 equipped with a 63x/1.40 Oil Plan-Apochromat objective and 488nm and 561 nm lasers (Carl Zeiss Microscopy, Göttingen, Germany). Images were acquired with a 1486 x 1486 pixel size and a bit depth of 16 bit. To quantify colocalization of CatB, CatL and NPC1 with Lamp-1-Scarlet, ten cells for each condition were selected and the Pearson Correlation Coefficient (PCC) for the overlap of CatB, CatL and NPC1-specific signals with Lamp-1 specific signals in single cells was determined using the Zeiss software ZEN 2.3 (blue edition). Solely for presentation purposes, confocal images were colored, converted to the JPEG format and cropped to 70% of their original size. No further image modifications were performed.

### Statistical analysis

Statistical analyses were performed by Welch’s t-test [58] for all transduction assays using Microsoft Excel 2013 and by Student’s t-test using Graphpad Prism 6 for the colocalization studies.

## Supporting information

S1 Fig Impact of SPP and SKI-1 inhibitor on processing of CatB and CatL. 293T cells were transfected with plasmids encoding CatB and CatL and incubated with the indicated concentrations of SKI-1 inhibitor (PF429242) or SPP inhibitor ((Z-LL)_2_-ketone) for the indicated times. Subsequently, CatB and CatL expression was analysed by immunoblot, using CatB and CatL specific antibodies. Expression of β-Actin (ACTB) served as negative control. Similar results were obtained in a separate experiment. CatB, cathepsin B; CatL, cathepsin L; PR, proform; UK, CatB/CatL fragment of unknown origin; SC, single chain; HC, heavy chain.
(PDF)
