# Supplementary material for: Inhibitors of signal peptide peptidase and subtilisin/kexin-isozyme 1 inhibit Ebola virus glycoprotein-driven cell entry by interfering with activity and cellular localization of endosomal cathepsins
Source: PLoS One. 2019 Apr 11;14(4):e0214968. doi: 10.1371/journal.pone.0214968 (PMC6459477; doi:10.1371/journal.pone.0214968)
Supplement: S1 Fig — 293T cells were transfected with plasmids encoding CatB and CatL and incubated with the indicated concentrations of SKI-1 inhibitor (PF429242) or SPP inhibitor ((Z-LL)2-ketone) for the indicated times. Subsequently, CatB and CatL expression was analysed by immunoblot, using CatB and CatL specific antibodies. Expression of β-Actin (ACTB) served as negative control. Similar results were obtained in a separate experiment. CatB, cathepsin B; CatL, cathepsin L; PR, proform; UK, CatB/CatL fragment of unknown origin; SC, single chain; HC, heavy chain. (PDF) [file pone.0214968.s001.pdf]

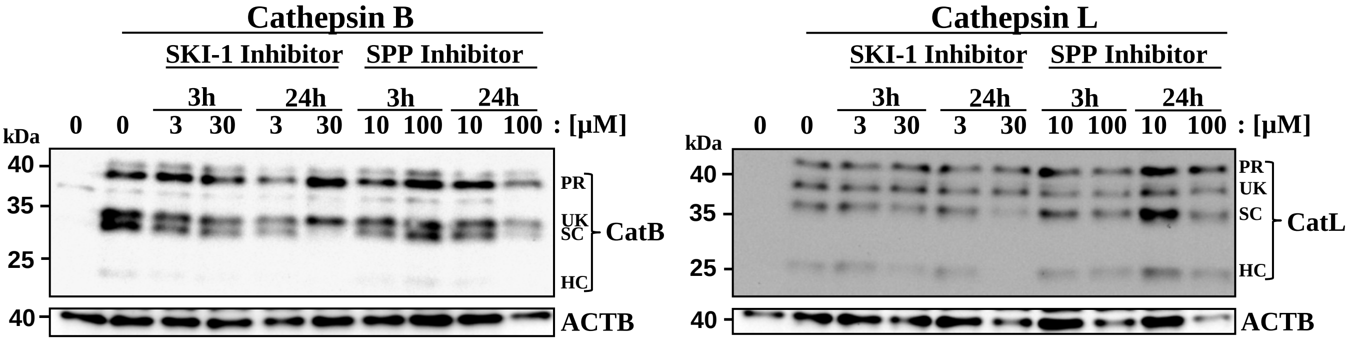

S1 Fig. Impact of SPP and SKI-1 inhibitor on processing of CatB and CatL. 293T cells were transfected with plasmids encoding CatB and CatL and incubated with the indicated concentrations of SKI-1 inhibitor (PF429242) or SPP inhibitor ((Z-LL)2-ketone) for the indicated times. Subsequently, CatB and CatL expression was analysed by immunoblot, using CatB and CatL specific antibodies. Expression of β-Actin (ACTB) served as negative control. Similar results were obtained in a separate experiment. CatB, cathepsin B; CatL, cathepsin L. PR, proform; UK, CatB/CatL fragment of unknown origin; SC, single chain; HC, heavy chain.
